# Primary bladder neck obstruction in females: Case series from the Indonesian population

**DOI:** 10.1016/j.ijscr.2024.110799

**Published:** 2024-12-30

**Authors:** Adelia Anggasta Adzhani, Tetuka Bagus Laksita, Johan Renaldo

**Affiliations:** Department of Urology, Faculty of Medicine, Airlangga University, Dr. Soetomo General Academic Hospital, Surabaya, Indonesia

**Keywords:** Primary bladder neck obstruction, PBNO, Transurethral resection of the bladder neck, Lower urinary tract symptoms, Urinary retention

## Abstract

**Introduction:**

Primary bladder neck obstruction (PBNO) is a rare but significant cause of BOO and LUTS in females, with unclear etiology involving theories of fibrotic narrowing, tissue hyperplasia, or muscle abnormalities. Due to nonspecific symptoms, PBNO diagnosis remains challenging, and optimal surgical treatment needs to be better defined.

**Case presentation:**

We report two cases of females in their 50s with recurrent urinary retention managed by indwelling catheters. The first, aged 58, experienced worsening LUTS over five months, requiring catheter use for two months. The second, aged 50, had persistent voiding difficulty and failed previous catheter trials. Both lacked a history of trauma, pelvic surgery, or significant comorbidities. Cystoscopy revealed bladder neck obstruction with moderate trabeculation of the detrusor muscle. Transurethral resection of the bladder neck (TURBN) was performed circumferentially. Postoperatively, both patients showed marked improvement, with uroflowmetry demonstrating maximum flow rates of 10.7 mL/s and 9.4 mL/s and minimal residual volumes. No incontinence or other complications were observed.

**Discussion:**

PBNO in females is underdiagnosed due to nonspecific presentations. Structural abnormalities and potential neurogenic factors are implicated in its pathophysiology. In our cases, TURBN effectively improved voiding function, underscoring its role for patients unresponsive to conservative therapy. However, data on long-term outcomes and risks, such as stress urinary incontinence, remain limited.

**Conclusion:**

TURBN is a promising treatment for female PBNO, offering symptom relief and improved voiding function. Continued research is needed to refine techniques and develop evidence-based guidelines.

## Highlights

• Primary bladder neck obstruction (PBNO) is a rare and challenging urologic condition in females, often requiring surgical intervention when conservative treatments fail.
• There is no consensus on the optimal surgical approach for transurethral resection of the bladder neck (TURBN) in female patients, highlighting the need for further research.
• This case series presents successful outcomes of TURBN in two female patients, demonstrating clinical improvement in uroflowmetry and symptom resolution.
• Surgical management of female PBNO must carefully consider the risk of urethral sphincter injury and potential incontinence, emphasizing the need for meticulous surgical technique.
• Our experience suggests that TURBN is a safe and effective treatment for resolving urinary retention in females with PBNO.

## 1 Introduction

Primary bladder neck obstruction (PBNO) is a rare cause of bladder outlet obstruction (BOO) and lower urinary tract symptoms (LUTS) in females [1]. The etiology of PBNO has yet to be fully understood, although several theories have been proposed regarding its underlying pathophysiology [2]. This condition is thought to result from narrowing caused by fibrous tissue or hyperplasia at the bladder neck [2]. Abnormal morphology of the detrusor/trigonal muscle arrangement is a potential cause of inefficient bladder neck opening [2]. The prevalence of female BOO varies widely, ranging from 2.7 % to 23 %, with the definition being variable and still controversial [3]. Data on the prevalence in females are limited, with most epidemiological studies reporting prevalence among sub-populations of females with BOO conditions [1]. The prevalence rate is estimated to range from 2.7 % to 8 % in females presenting with LUTS [4]. No studies have been published on PBNO in females in Indonesia.

Females with PBNO may experience a combination of LUTS, including bladder filling, voiding, and post-micturition symptoms, and some may have recurrent urinary tract infections. In certain cases, acute urinary retention has been reported in up to 16.6 % of patients [1]. The goals of treatment include protecting the upper urinary tract, managing infections, improving LUTS, and avoiding catheter or stoma dependence [3]. Surgical intervention in the form of transurethral resection of the bladder neck (TURBN) is generally recommended when conservative treatment fails to provide improvement. However, there currently needs to be a consensus on the optimal surgical technique for TURBN in female patients, and there is a need for more literature on PBNO in females in Indonesia. We describe our experience with PBNO in two female patients, highlighting the challenges and outcomes associated with surgical management through endourological approaches. This study has been reported using the SCARE (Surgical Case Report) criteria to ensure the quality and transparency of case reporting [5].

## 2 Case presentation

**Fig. 1:**
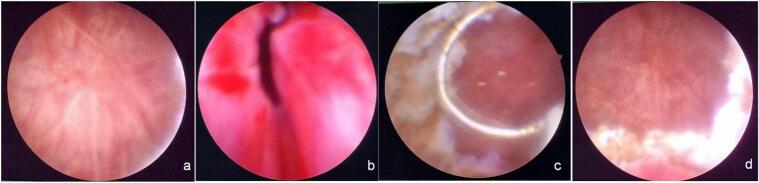
Images captured during the first patient's TURBN procedure. a (Trabeculation of the bladder wall), b (Bladder neck stenosis visualized during cystoscopy), c (TURBN in progress targeting the stenotic areas), d (Post procedure view of the bladder neck, demonstrating an open and unobstructed pathway following successful resection).

Two females in their 50s presented with persistent difficulties in spontaneous urination, requiring long-term catheterization. Both cases detail their ongoing urological symptoms, diagnostic findings, and subsequent treatment. The first case involves a 58-year-old woman with LUTS for five months, using a urethral catheter for the past two months. Her symptoms included difficulty voiding and straining during urination. She had no history of fever, hematuria, nephrolithiasis, trauma, pelvic surgery, diabetes, hypertension, or radiation exposure. Physical examination found a 16 French (Fr) urethral catheter with a 24-hour urine output between 1500 and 2000 mL. Urinalysis showed leukocytes and erythrocytes, indicating inflammation. Imaging, including an abdominal X-ray and ultrasound, showed no hydronephrosis or stones. Cystoscopy revealed moderate bladder wall trabeculation, “golf hole”-shaped ureteral orifices, and an elevated bladder neck. The patient underwent TURBN from the 3 to 9 o'clock direction up to the proximal external urethral sphincter, achieving bladder emptying using the Crede maneuver (Fig. 1). The catheter was removed the next day, and two weeks later, uroflowmetry indicated a maximum flow rate of 10.7 mL/s and a post-void residual (PVR) of 36 mL. Histopathology revealed chronic cystitis.

**Fig. 2:**
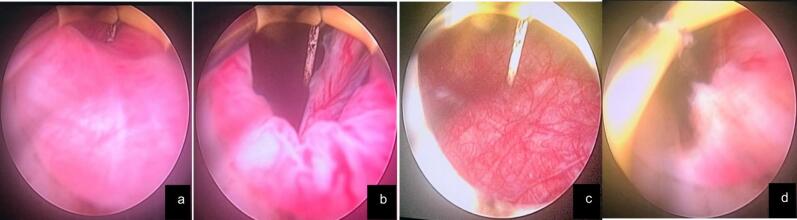
Images captured during the second patient's TURBN procedure. a and b (PBNO observed during cystoscopy), c (Trabeculation of the bladder wall), d (TURBN in progress, targeting the narrowed bladder neck tissue).

The second case involves a 50-year-old woman with a similar five-month history of voiding difficulties using a urethral catheter. Her symptoms included dysuria, incomplete bladder emptying, and a weak urine stream. She had previously undergone unsuccessful catheterization and cystoscopy with urethral dilation. Like the first patient, she had no history of fever, nephrolithiasis, or significant comorbidities. Examination found a 16-Fr catheter with a urine output of 1500–2000 mL/day. She was anemic, with hemoglobin at 9.7 mg/dL, but had normal creatinine levels. Urinalysis showed leukocyturia and hematuria. A non-contrast CT scan showed no stones or hydronephrosis. Cystoscopy revealed bladder trabeculation, cone-shaped ureteral orifices, and a thickened bladder neck. She also underwent TURBN from the 5 to 7 o'clock direction, with the catheter removed three days later (Fig. 2). Two weeks postoperatively, uroflowmetry showed a flow rate of 9.4 mL/s and a PVR of 22 mL. Histopathology confirmed chronic cystitis.

## 3 Discussion

PBNO is a functional bladder obstruction caused by abnormal bladder neck opening during the emptying phase of urination. It is a condition characterized by the absence of other anatomical causes or increased urethral sphincter activity, as described by Sussman, Drain, and Brucker [1]. Although it is typically considered a rare condition in females, it is an important cause of LUTS and can significantly impact the quality of life.

The cases reported in this study focus on two females in their 50s who presented with PBNO and were diagnosed via urethrocystoscopy, revealing a closed bladder neck. Patient selection was based on clinical presentations of persistent LUTS and BOO requiring long-term catheterization, with no history of trauma, pelvic surgery, diabetes, or other confounding conditions. The diagnosis was confirmed through cystoscopy, imaging, and uroflowmetry, excluding other causes, such as stones or malignancy. Both patients underwent TURBN as definitive treatment, with consistent postoperative evaluations, including uroflowmetry. These cases highlight the effectiveness of TURBN in managing PBNO in women, aligning with findings from previous studies.

BOO is often overlooked in females with LUTS. Although more common in men, it can present disruptive and bothersome symptoms in females, influencing their daily lives. The underlying etiology remains unclear, but various theories have been proposed. Early theories suggested structural changes in the bladder neck, such as fibrotic narrowing or tissue hyperplasia. Other hypotheses involve mesenchymal dissolution errors in the bladder neck, resulting in abnormal deposition of non-muscular tissue. Neurological etiologies related to sympathetic nervous system dysfunction have also been implicated [6].

The etiology remains elusive, though several theoretical frameworks have been proposed to explain its underlying mechanisms. One prominent theory suggests that PBNO arises from structural abnormalities, such as fibrotic tissue narrowing or hyperplasia at the bladder neck, which impede the normal opening mechanism required for effective voiding [6,7]. Histological investigations have revealed that mesenchymal cell dysfunction at the bladder neck may lead to the abnormal deposition of non-muscular tissues, contributing to these structural changes. Additionally, morphological abnormalities of the detrusor or trigonal muscles are implicated, potentially affecting the coordination required for bladder neck opening during the micturition phase [8]. Neurological factors have also been explored as contributors to PBNO. Dysregulation of the sympathetic nervous system, which governs bladder neck relaxation, may lead to functional obstructions even without structural anomalies [6,9]. This hypothesis aligns with clinical observations where patients exhibit voiding dysfunction without detectable anatomical abnormalities [9].

Skene's glands, also known as the paraurethral glands, are small glands located on either side of the urethral opening in females and are considered homologous to the male prostate. These glands are thought to secrete fluids that help lubricate the urethra and are involved in both urinary and reproductive health. Although typically small, Skene's glands can sometimes become inflamed or infected, leading to a condition known as Skene's gland cyst or abscess, which may contribute to urinary discomfort and obstruction. However, their exact function and potential role in urinary disorders still need to be completed and explored in clinical research. Currently, limited literature specifically addresses their role in the etiology of primary bladder neck obstruction. Given their anatomical proximity to the bladder neck, future research could explore whether pathological changes in Skene's glands, such as inflammation or hypertrophy, might contribute to bladder neck dysfunction or obstructive symptoms in females. This investigation could offer valuable insights and reveal new diagnostic or therapeutic approaches for PBNO in females, particularly for unresponsive cases to current treatments. Understanding this area might contribute to advancing knowledge about PBNO and female LUTS [10].

BOO in females can be categorized into two primary types: anatomical and functional. Clinical symptoms, medical history, and physical examination findings can often detect anatomical obstructions, such as incomplete bladder emptying or difficulties associated with pelvic organ prolapse (POP), urethral strictures, or post-surgical complications related to incontinence procedures. Functional obstructions, on the other hand, are identified during urination, as there are no apparent anatomical abnormalities associated with patient symptoms. The most common functional causes of BOO in neurologically normal females are dysfunctional voiding and PBNO. Dysfunctional voiding involves inappropriate relaxation or contraction of the external sphincter during urination. In contrast, PBNO involves failure of the proximal bladder neck to open and dilate properly [11]. Another condition, detrusor-external sphincter dyssynergia, is diagnosed based on known neurological disorders confirmed during urodynamic studies [12].

While primary bladder neck obstruction in females is globally recognized as rare, its study in the Indonesian context is particularly significant due to unique healthcare dynamics and potential population-specific factors. Indonesia's healthcare system faces challenges, including disparities in access to diagnostic and specialized urological services, particularly in rural and under-resourced areas, which may contribute to underdiagnosis and delayed treatment of such conditions [13]. Furthermore, cultural norms in Indonesia often discourage women from openly discussing urinary symptoms, potentially delaying medical consultations and interventions [14]. From an epidemiological perspective, environmental factors, such as exposure to tropical climates and dietary habits unique to the region, may play a role in bladder health and function. Additionally, genetic predispositions related to connective tissue or smooth muscle abnormalities could influence the prevalence of bladder-neck obstruction in this population. These factors still need to be explored and may differ significantly from those observed in Western populations, where most existing studies are concentrated [15]. This study contributes valuable insight into this condition within the Indonesian population, addressing a critical gap in the literature and highlighting the need for further research into how regional, environmental, and genetic factors might influence its development and management.

PBNO can lead to bladder outlet obstructions ranging from the bladder neck to the urethral meatus. This obstruction induces a varied and unpredictable bladder response, contributing to nonspecific LUTS without pathognomonic signs. Symptoms can include storage issues, voiding difficulties, post-micturition symptoms, and pelvic pain or discomfort [1]. In severe cases, patients may present with urinary retention [6]. Diagnosis involves a comprehensive clinical history and a thorough physical examination. Uroflowmetry and post-void residual volume (PVR) measurements are initial screening tools used in urological clinics to assess obstruction severity [1]. A pelvic examination is essential for females to evaluate for POP or other retention causes. In this series, neither uroflowmetry nor PVR tests were conducted due to a failed trial without a catheter (TWOC) in both patients. Initial treatment options for PBNO include alpha-blockers and surgical intervention on the bladder neck. While biofeedback physiotherapy is often ineffective due to high costs and time demands, surgical incision procedures, though invasive, are typically safe with minimal risk of incontinence [16].

TURBN has demonstrated promising results in the literature, providing definitive treatment for PBNO. A technique involving 5- and 7 o'clock incisions, with tissue resection between these points [3]. A median follow-up of three years revealed significant improvement in six out of seven patients, with only one experiencing mild stress urinary incontinence (SUI) [10]. Most procedures reported in the literature involve incisions at the 5 and 7 o'clock positions. In the presented cases, different surgical approaches (3 to 9 o'clock and 5 to 7 o'clock directions) were guided by anatomical considerations and the degree of bladder neck obstruction observed during cystoscopy. The 3 to 9 o'clock resection was chosen in the first case to address moderate trabeculation and ensure comprehensive relief of obstruction while preserving continence. In contrast, the second case's 5 to 7 o'clock approach was preferred due to the localized thickening of the bladder neck tissue in this region. These tailored approaches aimed to achieve optimal symptom relief while minimizing the risk of complications, such as stress urinary incontinence or damage to the external urethral sphincter. Shen et al. reported that making two deep incisions into the serosal layer resolved symptoms in 10 out of 11 females, with one patient experiencing mild SUI [17]. Another study by Peng and Kuo supported this approach, with similar success rates [18]. The TURBN procedure ensures that the external urethral sphincter remains intact, preserving continence [1]. Long-term follow-up is critical to monitor for complications or symptom recurrence. The procedure has improved Qmax, reduced PVR, and lowered the International Prostate Symptom Score (IPSS) in men [17].

Potential complications include urinary incontinence and vaginal wall perforation. Urinary incontinence, observed in approximately 6.2 % of patients, results from sphincter injury [19]. While the immediate postoperative outcomes of TURBN for PBNO appear favorable, long-term follow-up is essential to assess recurrence rates and potential complications, particularly SUI. Recurrence rates after TURBN vary between 10 % and 20 %, depending on factors such as the completeness of the initial procedure and the patient's underlying physiology [20]. Potential complications include urinary tract infections (UTIs), urethral strictures, vaginal wall perforation, and de novo or worsening SUI. Vaginal wall perforation, though rare, has been reported in 1–2 % of cases, primarily related to excessive incision depth [21]. Urethral strictures may develop in 5–10 % of patients due to scar formation at the surgical site [22]. Long-term follow-up should include periodic evaluations through uroflowmetry, PVR measurement, and symptom assessment based on patient-reported outcomes to monitor for obstructive symptoms or functional decline. Regular pelvic examinations and cystoscopy assessments may also help detect anatomical changes or scarring at the surgical site. In cases of recurrence, repeat TURBN may be considered to restore bladder function. Additionally, attention should be given to the potential for developing SUI, which, although reported in approximately 6 % of cases, could significantly impact quality of life [23]. For patients experiencing SUI, pelvic floor physiotherapy and, in severe cases, surgical interventions such as mid-urethral sling procedures may be necessary to restore continence. Standardized follow-up protocols and further longitudinal studies are needed to optimize patient outcomes and refine strategies for preventing recurrence and complications.

Patient-reported outcomes provide critical insights into the real-world impact of surgical interventions. In our cases, both patients reported significant relief from symptoms, particularly the psychological and physical burdens associated with catheter dependence. They described improved daily functioning and increased confidence in social and occupational settings. These observations align with the objective improvements observed postoperatively, underscoring the holistic benefits of TURBN for PBNO management. Future studies should incorporate standardized patient-reported outcome measures to quantify these aspects better and refine care strategies.

## 4 Conclusion

The occurrence of PBNO in females is rare in everyday clinical practice, with management options ranging from conservative to surgical interventions. The primary aim of treating PBNO is to prevent complications and improve female urinary function. Endourological resection has proven effective in many cases; however, limited literature on bladder neck resection procedures specifically for PBNO in females still needs to be improved. Further research and publications are needed to enhance understanding and develop comprehensive guidelines for managing PBNO in this population.

## Author contribution

Adelia Anggasta Adzhani: Investigation, Methodology, Resources, Writing - original draft, Conceptualization, Supervision, Project administration, Writing - review & editing.

Tetuka Bagus Laksita: Conceptualization, Methodology, Writing - original draft, Writing - review & editing.

Johan Renaldo: Conceptualization, Methodology, Writing - original draft, Writing - review & editing.

## Consent

Written informed consent was obtained from the patient for publication of this case report and accompanying images. A copy of the written consent is available for review by the Editor-in-Chief of this journal on request.

## Ethical approval

Ethical approval to report this case was obtained from The Hospital Research Ethics Committee of “Rumah Sakit Umum Dokter Soetomo Surabaya” where the patient was admitted.

## Guarantor

Adelia Anggasta Adzhani.

## Research registration number

Not required.

## Funding

The authors received no financial support for the research, authorship and/or publication of this article.

## Declaration of competing interest

The authors report no declarations of interest.
